# Supplementary material for: Coexistence of Dominant Marine Phytoplankton Sustained by Nutrient Specialization
Source: Microbiol Spectr. 2023 Jul 17;11(4):e04000-22. doi: 10.1128/spectrum.04000-22 (PMC10441275; doi:10.1128/spectrum.04000-22)
Supplement: Supplemental file 2 — Supplemental material. Download spectrum.04000-22-s0001.pdf, PDF file, 1.9 MB [file spectrum.04000-22-s0001.pdf]

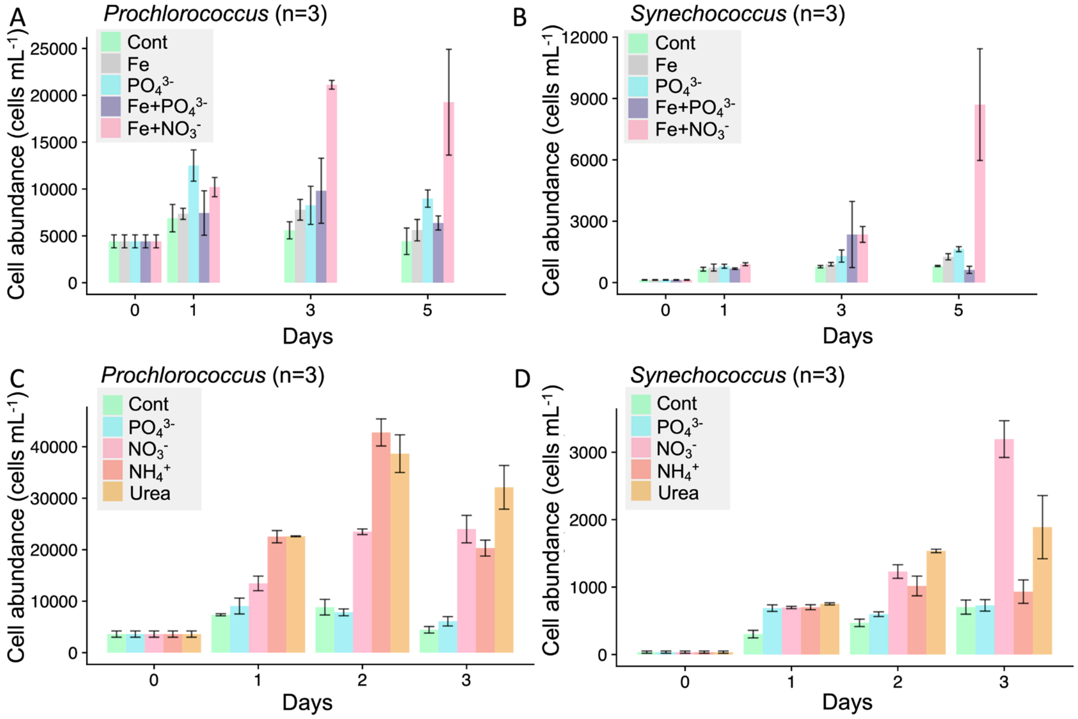

Fig. S1 Effect of nutrient amendment on *Prochlorococcus* and *Synechococcus* abundance. (A) and (B) The effect of Fe and P additions on cell abundance (means ± SD, n = 3) for Exp. Fe2. Addition of Fe and P had no effect on the growth of *Prochlorococcus* and *Synechococcus* except when N was also added. (C) and (D) The effect of different chemical forms of N and P additions on cell abundance (means ± SD, n = 3) for Exp. M3. *Prochlorococcus* grows better with NH_4^+ and urea while *Synechococcus* grows better with NO_3^-. Cont; control.

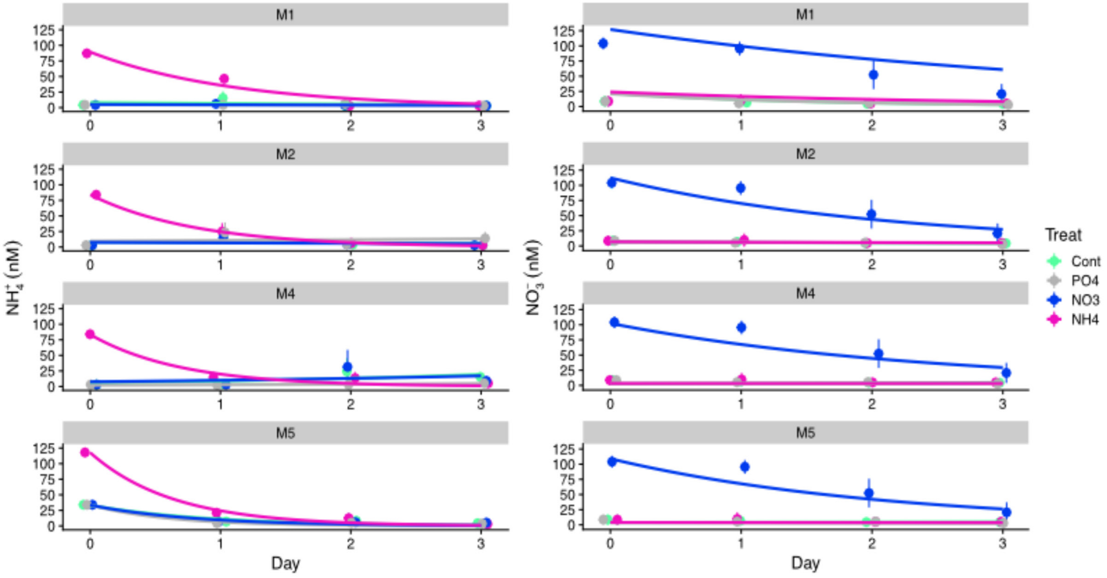

Fig. S2 Temporal change in NH_4^+ and NO_3^- concentrations of Ex. M1, M2, M4 and M5. (A) NH_4^+ concentration in the NH_4^+ treatment exponentially decreased during the experiment down to the detection limit of 6 nM on day 3. (B) NO_3^- concentrations in the NO_3^- treatment exponentially decreased during the experiment but enriched NO_3^- was not always entirely consumed. Error bar shows a standard deviation of triplicate.

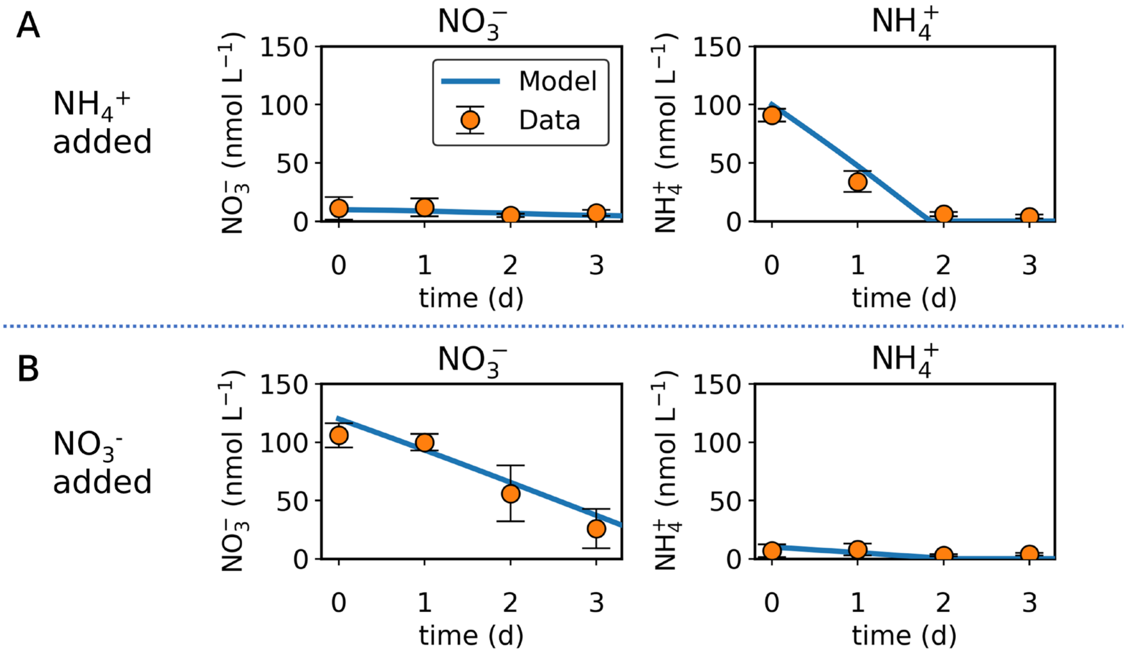

Fig. S3 Model-data comparison of the time series of NH_4^+ and NO_3^- concentrations. (A) NH_4^+ added. (B) NO_3^- added. Points, Data; Curves, Model. Data are based on Exp. M1-M3. Sample numbers for each data are same as Fig. 1. The error bars represent the standard deviation based on the mean values across from the selected experiments.

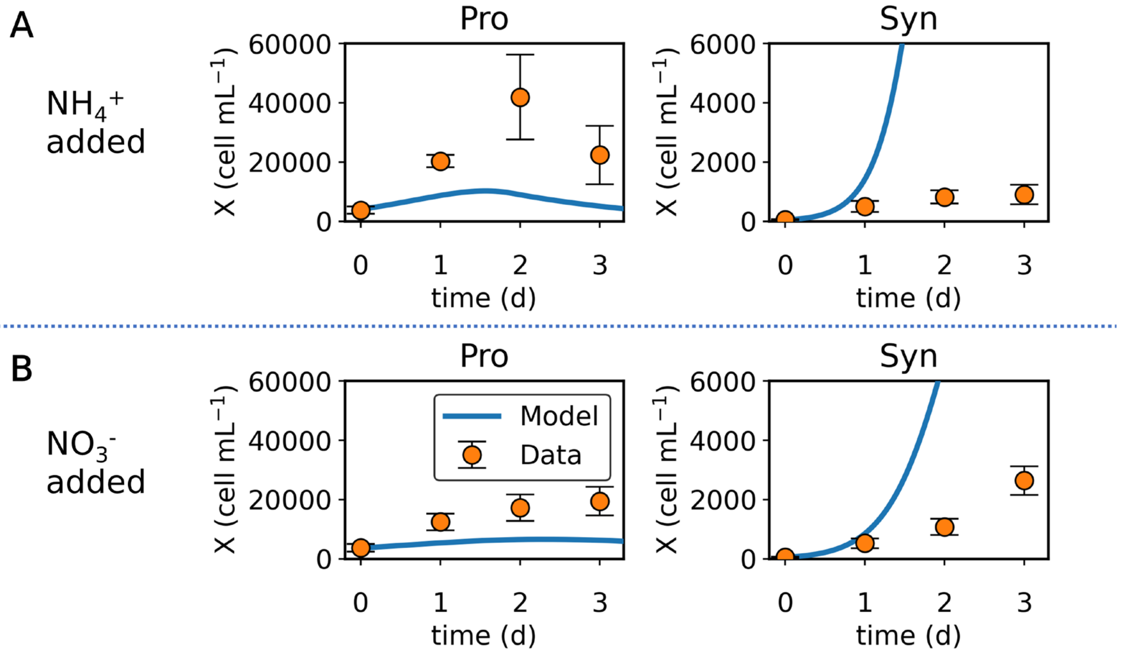

Fig. S4 Model-data comparison of the time series of abundance (X) of *Prochlorococcus* and *Synechococcus* with the K_{NH4} values flipped between these organisms. (A) NH_4^+ added. (B) NO_3^- added. Points, Data; Curves, Model. Pro, *Prochlorococcus*; Syn, *Synechococcus*. Data are the same as Fig. 1.

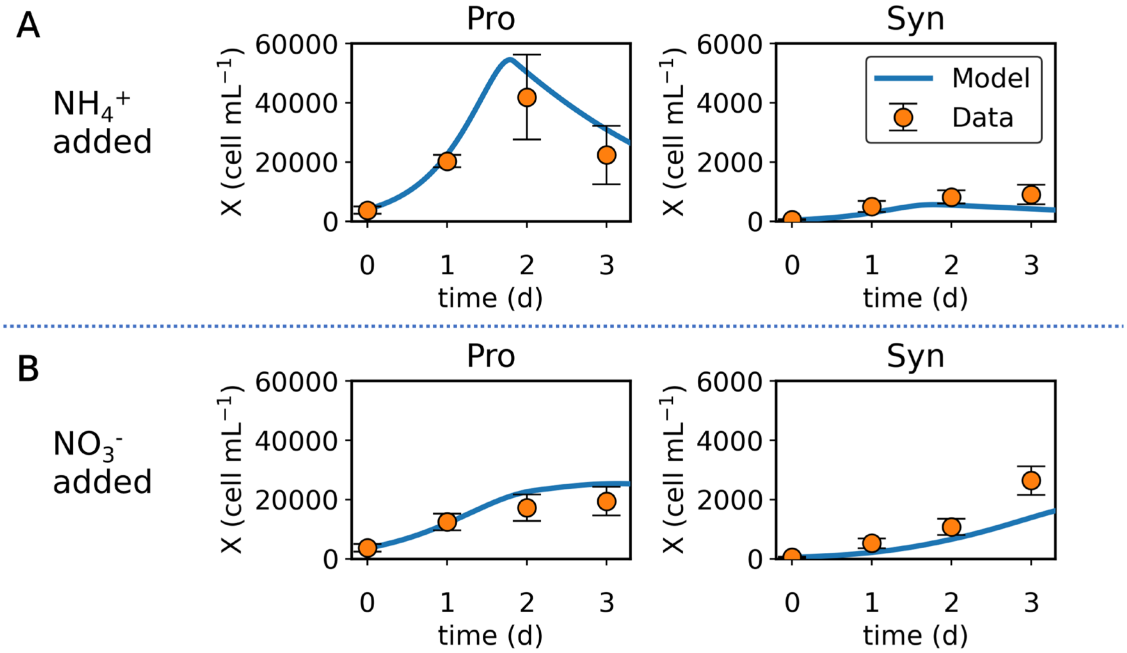

Fig. S5 Model-data comparison of the time series of abundance (*X*) of *Prochlorococcus* and *Synechococcus* with the same high K_{NO3} values for these organisms. (A) NH_4^+ added. (B) NO_3^- added. Points, Data; Curves, Model. Pro, *Prochlorococcus*; Syn, *Synechococcus*. Data are based on Exp. M1-M3. We applied K_{NO3} of *Synechococcus* to *Prochlorococcus*. Data are the same as Fig. 1.

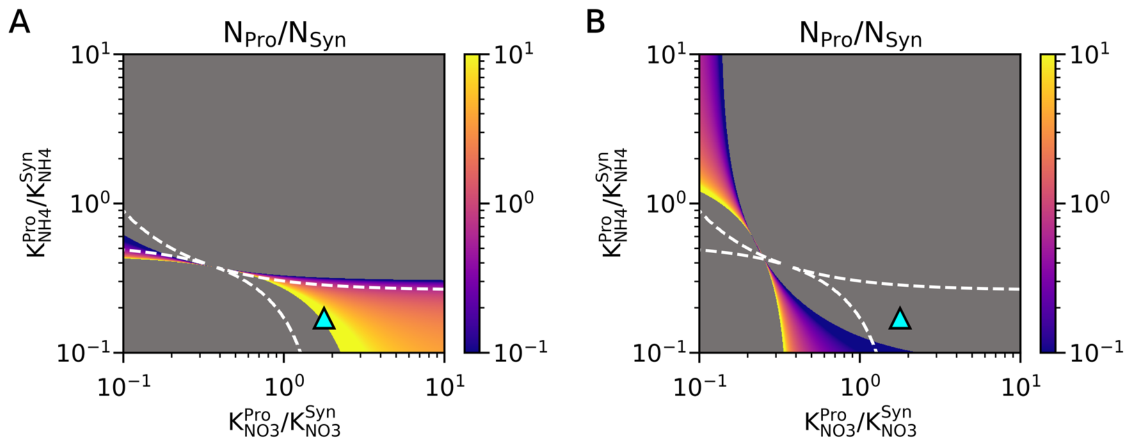

Fig. S6 The effect of the change in the resource ratio. (A) NO_3^-:NH_4^+ = 1:6 (B) NO_3^-:NH_4^+ = 3:1. The cyan triangle represents the ratios of predicted half-saturation constants for Figure 2. Dashed curves indicate borders between coexistence and competitive exclusion when NO_3^-:NH_4^+ = 3:1 (same dashed curves as in Figure 4A).

Table S1 The degrees of freedom (df), *F*-values and *p*-values of the two-way RM-ANOVA for cell density (Fig. 1). Day represents the timing of harvest (1, 2, 3 for Exp. M1-5, 1, 3 for Exp. Fe1-3); Treatment represents the enriched nutrient (Cont, PO_4^{3-}, NO_3^-, NH_4^+, Urea for Exp. M1-5, Cont, PO_4^{3-}, Fe, Fe + PO_4^{3-}, Fe + NO_3^- for Exp. Fe1-3). RM-ANOVA shows that cell densities of both *Prochlorococcus* and *Synechococcus* were different among treatments.

		df	*F*-value	*p*-value	df	*F*-value	*p*-value
Exp.			*Prochlorococcus*			*Synechococcus*	
M1	Day	2	3.625	>0.05	2	24.332	<0.01
	Treatment	4	8.6115	<0.01	4	6.080	<0.01
	Day × Treatment	8	4.160	0.01<*p*<0.05	8	2.289	0.01<*p*<0.05
M2	Day	2	7.762	<0.01	2	19.022	<0.01
	Treatment	4	39.875	<0.01	4	84.280	<0.01
	Day × Treatment	8	3.332	<0.01	8	27.309	<0.01
M3	Day	2	28.584	<0.01	2	36.558	<0.01
	Treatment	4	80.641	<0.01	4	30.091	<0.01
	Day × Treatment	8	8.872	<0.01	8	11.662	<0.01
M4	Day	2	7.040	<0.01	2	12.845	<0.01
	Treatment	4	39.211	<0.01	4	57.375	<0.01
	Day × Treatment	8	5.856	<0.01	8	5.475	<0.01
M5	Day	2	36.767	<0.01	2	135.416	<0.01
	Treatment	4	29.780	<0.01	4	94.340	<0.01
	Day × Treatment	8	3.831	<0.01	8	37.498	<0.01
Fe1	Day	2	38.127	<0.01	2	30.059	<0.01
	Treatment	4	87.178	<0.01	4	34.875	<0.01
	Day × Treatment	8	33.909	<0.01	8	10.743	<0.01
Fe2	Day	2	23.810	<0.01	2	67.473	<0.01
	Treatment	4	6.027	<0.01	4	7.789	<0.01
	Day × Treatment	8	6.027	<0.01	8	7.789	<0.01
Fe3	Day	2	61.100	<0.01	2	34.409	<0.01
	Treatment	4	7.114	<0.01	4	4.460	0.01<*p*<0.05
	Day × Treatment	8	7.114	<0.01	8	4.460	0.01<*p*<0.05

Table S2 The initial physical and chemical conditions of seawater collected for each bioassay experiment. For all data, means are shown with ± standard deviation for triplicate samples. ND, no data. SRP; soluble reactive phosphorus, TFe; total iron, DFe; dissolved iron (< 0.22 μm), Pro.; Prochlorococcus, Syn.; Synechococcus, Cro.; Crocosphaera, PicoE; Pico-eukaryotes.

Exp.	Date In 2008	Chl a (ng L^{-1})	Salinity	Temperature (ºC)	NO_3^-+NO_2^- (nM)	NH4+ (nM)	SRP (nM)	TFe (nM)	DFe (nM)	Pro. (cells mL^{-1})	Syn. (cells mL^{-1})	Cro. (cells mL^{-1})	PicoE. (cells mL^{-1})
M1	6 June	24	34.27	29.0	<3	<3	56	0.34	0.11	5275 ± 1628	70 ± 5	32 ± 62	366 ± 118
M2	10 June	25	34.19	29.3	<3	<3	51	0.29	0.17	2235 ± 226	38 ± 3	129	614 ± 72
M3	14 June	28	34.24	29.1	<3	9	64	ND	ND	3609 ± 1051	35 ± 29	126 ± 32	410 ± 137
M4	18 June	61	34.13	29.1	<3	15	31	0.47	0.44	26212 ± 2227	1280 ± 131	1513 ± 684	804 ± 44
M5	22 June	98	33.94	28.9	7	36	31	1.01	0.16	26767 ± 3662	522 ± 101	306 ± 112	553 ± 54

Table S3 Summary of the effects of nutrient additions on cell density in each bioassay experiment. Cell density was measured from separate triplicate bottles. The response of the phytoplankton communities to the different treatments was compared by post hoc Tukey test, to compare the means between five treatments (n = 3 replicates per treatment throughout, degrees of freedom = 40). Significant differences ($p < 0.05$) between individual treatments are indicated by <; = indicates no significant differences. Cont, PO4, NO3, NH4, Urea refer to the treatment of control, PO_4^{3+}, NO_3^-, NH_4^+ and urea respectively. ND refers to no significant difference. Tukey test shows that *Prochlorococcus* prefers recycled N, such as NH_4^+ and urea while *Synechococcus* prefer NO_3^-.

Exp.	Day	*Prochlorococcus*	*Synechococcus*
M1	1	Cont = PO4 = NO3 = NH4 = Urea	Cont = PO4 = NO3 = NH4 = Urea
	2	(Cont = PO4 = NO3) < (NH4 = Urea)	(Cont = PO4 = Urea) < NO3 < NH4
	3	(Cont = PO4) < (NO3 = Urea) < NH4	Cont = PO4 = NO3 = NH4 = Urea
M2	1	(Cont = PO4 = NO3) < NH4 < Urea	Cont = PO4 = NO3 = NH4 = Urea
	2	(Cont = PO4 = NO3) < NH4 < Urea	(Cont = PO4 = NH4) < NO3 = Urea
	3	(Cont = PO4 = NO3 = NH4) < Urea	(Cont = PO4 = NH4 = Urea) < NO3
M3	1	(Cont = PO4) < NO3 < Urea < NH4	Cont < (PO4 = NO3= NH4 = Urea)
	2	(Cont = PO4) < NO3 < (NH4 = Urea)	(Cont = PO4) = NH4 < NO3 < Urea
	3	(Cont = PO4) < NH4 < NO3 < Urea	(Cont = PO4 = NH4 = Urea) < NO3
M4	1	Cont < PO4 < (NO3 = Urea) < NH4	PO4 < (Cont = NO3 = NH4 = Urea)
	2	(Cont = PO4) < NO3 < Urea < NH4	PO4 < Urea < (Cont = NH4) < NO3
	3	(Cont = PO4) < Urea < NH4 < NO3	PO4 < Urea < (Cont = NH4) < NO3
M5	1	PO4 < (Cont = NO3 = NH4 = Urea)	Cont = PO4 = NO3 = NH4 = Urea
	2	(Cont = PO4) < NO3 < Urea < NH4	Cont = PO4 = NO3 = NH4 = Urea
	3	(Cont = PO4) < (NO3 = NH4) < Urea	Cont = PO4 = NO3 = NH4 = Urea
Fe1	1	Cont = Fe = PO4 = Fe+PO4 = Fe+NO3	Cont = Fe = PO4 = Fe+PO4 = Fe+NO3
	3	(Cont = Fe = PO4 = Fe+PO4) <Fe+NO3	(Cont = Fe = PO4 = Fe+PO4) <Fe+NO3
	5	(Cont = Fe = PO4 = Fe+PO4) <Fe+NO3	(Cont = Fe = PO4 = Fe+PO4) <Fe+NO3
Fe2	1	Cont = Fe = PO4 = Fe+PO4 = Fe+NO3	Cont = Fe = PO4 = Fe+PO4 = Fe+NO3
	3	(Cont = Fe = PO4 = Fe+PO4) <Fe+NO3	(Cont = Fe = PO4 = Fe+PO4) <Fe+NO3
	5	(Cont = Fe = PO4 = Fe+PO4) <Fe+NO3	(Cont = Fe = PO4 = Fe+PO4) <Fe+NO3
Fe3	1	Cont = Fe = PO4 = Fe+PO4 = Fe+NO3	Cont = Fe = PO4 = Fe+PO4 = Fe+NO3
	3	(Cont = Fe = PO4 = Fe+PO4) <Fe+NO3	(Cont = Fe = PO4 = Fe+PO4) <Fe+NO3
	5	(Cont = Fe = PO4 = Fe+PO4) <Fe+NO3	(Cont = Fe = PO4 = Fe+PO4) <Fe+NO3

Table S4 Parameter values for the model.

Parameter	Value	Unit
V_{maxNO3}^{Pro}	1.14	d^{-1}
V_{maxNH4}^{Pro}	2.42	d^{-1}
V_{maxNO3}^{Syn}	2.17	d^{-1}
V_{maxNH4}^{Syn}	3.98	d^{-1}
K_{NO3}^{Pro}	35.5	nmol L^{-1}
K_{NH4}^{Pro}	11.8	nmol L^{-1}
K_{NO3}^{Syn}	19.9	nmol L^{-1}
K_{NH4}^{Syn}	69.2	nmol L^{-1}
m_{Pro}	0.706	d^{-1}
m_{Syn}	0.517	d^{-1}
Q_N^{Pro}	*1.20×10^{-4}	nmol $cell^{-1}$
Q_N^{Syn}	*2.88×10^{-4}	nmol $cell^{-1}$
NH_4^+ added case		
S_{NO3}	-1	nmol L^{-1} d^{-1}
S_{NH4}	-50	nmol L^{-1} d^{-1}
NO_3^- added case		
S_{NO3}	-26	nmol L^{-1} d^{-1}
S_{NH4}	-4	nmol L^{-1} d^{-1}

*Average values based on Exp. M1-M3.

Table S5 Calculated affinity values

Parameter	Value	Unit
$V_{maxNO3}^{Pro}/K_{NO3}^{Pro}$	0.0322	d^{-1} $nmol^{-1}$ L
$V_{maxNH4}^{Pro}/K_{NH4}^{Pro}$	0.2053	d^{-1} $nmol^{-1}$ L
$V_{maxNO3}^{Syn}/K_{NO3}^{Syn}$	0.1086	d^{-1} $nmol^{-1}$ L
$V_{maxNH4}^{Syn}/K_{NH4}^{Syn}$	0.0575	d^{-1} $nmol^{-1}$ L

Table S6 Initial values for the simulated time series

Parameter	Value	Unit
NH_4^+ added case		
X_{Pro}	4.00×10^6	cell L^{-1}
X_{Syn}	4.00×10^4	cell L^{-1}
$[NO_3^-]$	10	nmol L^{-1}
$[NH_4^+]$	100	nmol L^{-1}
NO_3^- added case		
X_{Pro}	3.60×10^6	cell L^{-1}
X_{Syn}	4.80×10^4	cell L^{-1}
$[NO_3^-]$	120	nmol L^{-1}
$[NH_4^+]$	10	nmol L^{-1}

Table S7 CMIP5 Earth System Models used in this study. For future climate projections, all model output was from the RCP8.5 emissions scenario and was downloaded from: https://esgf-node.llnl.gov/search/cmip5/

**Modeling Center (or Group)**	**Model Name**	**Nutrient**
Community Earth System Model Contributors, NCAR	CESM1 (BGC)	NO_3^-
NOAA Geophysical Fluid Dynamics Laboratory	GFDL-ESM2M	NO_3^-
Met Office Hadley Centre (additional HadGEM2-ES realizations contributed by Instituto Nacional de Pesquisas Espaciais)	HadGEM2-ES	NO_3^-
Institut Pierre-Simon Laplace	IPSL-CM5A-LR	NO_3^-
Max-Planck-Institut für Meteorologie (Max Planck Institute for Meteorology)	MPI-ESM-LR	NO_3^-

Table S8 List of strains and genome assemblies used for phylogenomic reconstruction in Figure 6, and their placement in clades as depicted therein.

Strain/Isolate (Name in GenBank)	GenBank Assembly Accession	Clade
Synechococcus elongatus PCC 11801	GCA_003846445.1	*Synechococcus* sensu stricto
Synechococcus elongatus PCC 6301	GCA_022984195.1	*Synechococcus* sensu stricto
Aphanocapsa feldmannii 277cV	GCA_004768415.1	*Aphanocapsa feldmannii*
Candidatus Synechococcus spongiarum LMB bulk15M	GCA_002018045.1	*candidatus Synechococcus spongiarum = Synechospogium*
Candidatus Synechococcus spongiarum SH4	GCA_000586015.1	*candidatus Synechococcus spongiarum = Synechospogium*
Candidatus Synechococcus spongiarum SP3	GCA_001007665.1	*candidatus Synechococcus spongiarum = Synechospogium*
Candidatus Synechococcus spongiarum m9	GCA_900047545.1	*candidatus Synechococcus spongiarum = Synechospogium*
Synechococcus lacustris Tous	GCA_003011125.1	*Lacustricoccus lacustris*
Synechococcus sp. RCC307	GCA_000063525.1	*Immanicoccus*
Synechococcus sp. TMED19	GCA_002168155.1	*Immanicoccus*
Cyanobacteria bacterium UBA7373	GCA_002470305.1	*Immanicoccus*
Synechococcus sp. MW101C3	GCA_002252635.1	*Cyanobium*/*Regnicoccus*/*Vulcanococcus*
Synechococcus sp. WH 5701	GCA_000153045.1	*Cyanobium*/*Regnicoccus*/*Vulcanococcus*
Synechococcus sp. 1G10	GCA_002252625.1	*Cyanobium*/*Regnicoccus*/*Vulcanococcus*
Cyanobium sp. ULC084bin3	GCA_003249115.1	*Cyanobium*/*Regnicoccus*/*Vulcanococcus*
Synechococcus sp. SynAce01	GCA_001885215.1	*Cyanobium*/*Regnicoccus*/*Vulcanococcus*

Synechococcus sp. Baikal-G1	GCA_002737305.1	*Cyanobium*/*Regnicoccus*/*Vulcanococcus*
Vulcanococcus limneticus LL	GCA_002252705.1	*Cyanobium*/*Regnicoccus*/*Vulcanococcus*
Synechococcus sp. CB0101	GCA_000179235.2	*Cyanobium*/*Regnicoccus*/*Vulcanococcus*
Synechococcus sp. CB0205	GCA_000179255.1	*Cyanobium*/*Regnicoccus*/*Vulcanococcus*
Cyanobium sp. CACIAM 14	GCA_000708525.1	*Cyanobium*/*Regnicoccus*/*Vulcanococcus*
Synechococcus sp. BO 8801	GCA_002252675.1	*Cyanobium*/*Regnicoccus*/*Vulcanococcus*
Cyanobium gracile PCC 6307	GCA_000316515.1	*Cyanobium*/*Regnicoccus*/*Vulcanococcus*
Aphanothece cf. *minutissima* CCALA 015	GCA_003003925.1	*Cyanobium*/*Regnicoccus*/*Vulcanococcus*
Synechococcus sp. 8F6	GCA_002252665.1	*Cyanobium*/*Regnicoccus*/*Vulcanococcus*
Cyanobium usitatum str. Tous	GCA_003011885.1	*Cyanobium*/*Regnicoccus*/*Vulcanococcus*
Synechococcus sp. GFB01	GCA_001039265.1	*Cyanobium*/*Regnicoccus*/*Vulcanococcus*
Cyanobacteria bacterium UBA5018	GCA_002396505.1	*Cyanobium*/*Regnicoccus*/*Vulcanococcus*
Cyanobium sp. PCC 7001	GCA_000155635.1	*Cyanobium*/*Regnicoccus*/*Vulcanococcus*
Cyanobium sp. NIES-981	GCA_900088535.1	*Cyanobium*/*Regnicoccus*/*Vulcanococcus*
Cyanobium sp. CSSed165cm_191R1	GCA_007135755.1	*Cyanobium*/*Regnicoccus*/*Vulcanococcus*
Synechococcus sp. KORDI-49	GCA_000737575.1	*Parasynechococcus*
Synechococcus sp. MED850	GCA_002700765.1	*Parasynechococcus*
Synechococcus sp. CC9616	GCA_000515235.1	*Parasynechococcus*
Synechococcus sp. KORDI-100	GCA_000737535.1	*Parasynechococcus*
Cyanobium sp. NAT70	GCA_002701375.1	*Parasynechococcus*
Synechococcus sp. AG-670-F04	GCA_003211515.1	*Parasynechococcus*
Synechococcus sp. CPC35	GCA_002684175.1	*Parasynechococcus*

Synechococcus sp. AG-683-C23	GCA_003208835.1	*Parasynechococcus*
Synechococcus sp. CC9902	GCA_000012505.1	*Parasynechococcus*
Synechococcus sp. BL107	GCA_000153805.1	*Parasynechococcus*
Synechococcus sp. MED650	GCA_002691345.1	*Parasynechococcus*
Synechococcus sp. UW69	GCA_900474185.1	*Parasynechococcus*
Synechococcus sp. AG-670-B23	GCA_003209165.1	*Parasynechococcus*
Synechococcus sp. KORDI-52	GCA_000737595.1	*Parasynechococcus*
Synechococcus sp. CC9605	GCA_000012625.1	*Parasynechococcus*
Synechococcus sp. WH 8109	GCA_000161795.2	*Parasynechococcus*
Synechococcus sp. UW86	GCA_900474085.1	*Parasynechococcus*
Synechococcus sp. AG-673-B04	GCA_003211235.1	*Parasynechococcus*
Synechococcus sp. AG-679-D13	GCA_003210735.1	*Parasynechococcus*
Synechococcus sp. AG-679-B05	GCA_003210795.1	*Parasynechococcus*
Synechococcus sp. WH 8102	GCA_000195975.1	*Parasynechococcus*
Synechococcus sp. MED-G133	GCA_004212765.1	*Parasynechococcus*
Synechococcus sp. AG-670-A05	GCA_003211535.1	*Parasynechococcus*
Cyanobacteria bacterium TMED177	GCA_002170825.1	*Parasynechococcus*
Synechococcus sp. ARS1019	GCA_002690325.1	*Parasynechococcus*
Synechococcus sp. RS9916	GCA_000153825.1	*Pseudosynechococcus*
Synechococcus sp. UW105	GCA_900473935.1	*Pseudosynechococcus*
Synechococcus sp. UW140	GCA_900474295.1	*Pseudosynechococcus*
Synechococcus sp. BS55D	GCA_004332415.1	*Pseudosynechococcus*
Synechococcus sp. RS9917	GCA_000153065.1	*Pseudosynechococcus*

Synechococcus sp. WH 8101	GCA_00420977 5.1	*Pseudosynechococcus*
Synechococcus sp. WH 7803	GCA_00006350 5.1	*Pseudosynechococcus*
Synechococcus sp. TMED90	GCA_00217293 5.1	*Pseudosynechococcus*
Synechococcus sp. WH 7805	GCA_00015328 5.1	*Pseudosynechococcus*
Synechococcus sp. EAC657	GCA_00269328 5.1	*Pseudosynechococcus*
Cyanobium sp. ARS6	GCA_002687115 .1	*Pseudosynechococcus*
Synechococcus sp. AG-679-A04	GCA_00321077 5.1	*Pseudosynechococcus*
Synechococcus sp. AG-673-F03	GCA_003211205 .1	*Pseudosynechococcus*
Synechococcus sp. AG-670-F22	GCA_00320915 5.1	*Pseudosynechococcus*
Synechococcus sp. MIT S9508	GCA_00163216 5.1	*Pseudosynechococcus*
Synechococcus sp. MIT S9509	GCA_00163193 5.1	*Pseudosynechococcus*
Synechococcus sp. UW179A	GCA_90047396 5.1	*Pseudosynechococcus*
Synechococcus sp. AG-679-C18	GCA_00321075 5.1	*Pseudosynechococcus*
Synechococcus sp. AG-686-F08	GCA_00321031 5.1	*Pseudosynechococcus*
Synechococcus sp. AG-683-A03	GCA_00321055 5.1	*Pseudosynechococcus*
Synechococcus sp. CC9311	GCA_00001458 5.1	*Pseudosynechococcus*
Synechococcus sp. WH 8020	GCA_00104084 5.1	*Pseudosynechococcus*
Synechococcus sp. MED-G68	GCA_00333178 5.1	*Pseudosynechococcus*
Synechococcus sp. WH 8016	GCA_00023067 5.2	*Pseudosynechococcus*
Prochlorococcus sp. MIT 0701	GCA_00076029 5.1	*Thaumococcus*, LL IV
Prochlorococcus sp. AG-670-J21	GCA_003211485 .1	*Thaumococcus*, LL IV
Prochlorococcus marinus str. MIT 9313	GCA_000011485 .1	*Thaumococcus*, LL IV

Prochlorococcus sp. AG-409-P01	GCA_003215335.1	unassigned *Prochlorococcus* clade
Prochlorococcus sp. AG-363-B04	GCA_003278465.1	unassigned *Prochlorococcus* clade
Prochlorococcus sp. AG-363-O16	GCA_003280095.1	unassigned *Prochlorococcus* clade
Prochlorococcus sp. AG-363-J23	GCA_003280245.1	unassigned *Prochlorococcus* clade
Prochlorococcus sp. AG-409-A19	GCA_003216155.1	unassigned *Prochlorococcus* clade
Prochlorococcus sp. AG-409-J16	GCA_003215655.1	unassigned *Prochlorococcus* clade
Prochlorococcus sp. AG-409-N21	GCA_003215415.1	unassigned *Prochlorococcus* clade
Prochlorococcus sp. AG-409-J19	GCA_003215635.1	unassigned *Prochlorococcus* clade
Prochlorococcus sp. AG-409-M05	GCA_003215435.1	unassigned *Prochlorococcus* clade
Prochlorococcus sp. AG-363-P19	GCA_003280055.1	unassigned *Prochlorococcus* clade
Prochlorococcus sp. AG-363-L02	GCA_003280185.1	unassigned *Prochlorococcus* clade
Prochlorococcus sp. AG-363-M17	GCA_003278365.1	unassigned *Prochlorococcus* clade
Prochlorococcus sp. AG-363-P08	GCA_003278195.1	unassigned *Prochlorococcus* clade
Prochlorococcus sp. AG-409-B05	GCA_003216075.1	unassigned *Prochlorococcus* clade
Prochlorococcus sp. AG-363-O15	GCA_003278255.1	unassigned *Prochlorococcus* clade
Prochlorococcus sp. AG-409-B13	GCA_003216035.1	unassigned *Prochlorococcus* clade
Prochlorococcus sp. AG-409-O23	GCA_003215355.1	unassigned *Prochlorococcus* clade
Prochlorococcus sp. AG-409-L18	GCA_003215535.1	unassigned *Prochlorococcus* clade
Prochlorococcus sp. AG-409-G20	GCA_003215775.1	unassigned *Prochlorococcus* clade
Prochlorococcus sp. AG-409-L14	GCA_003215545.1	unassigned *Prochlorococcus* clade
Prochlorococcus sp. AG-463-P14	GCA_003209275.1	unassigned *Prochlorococcus* clade
Prochlorococcus sp. AG-363-O06	GCA_003280085.1	unassigned *Prochlorococcus* clade
Prochlorococcus sp. AG-409-P03	GCA_003215295.1	unassigned *Prochlorococcus* clade

Prochlorococcus sp. AG-363-A16	GCA_003280345.1	unassigned *Prochlorococcus* clade
Prochlorococcus sp. AG-363-N20	GCA_003280105.1	unassigned *Prochlorococcus* clade
Prochlorococcus sp. AG-363-K07	GCA_003280225.1	unassigned *Prochlorococcus* clade
Prochlorococcus sp. AG-463-F15	GCA_003211755.1	unassigned *Prochlorococcus* clade
Prochlorococcus sp. AG-363-C20	GCA_003280295.1	unassigned *Prochlorococcus* clade
Prochlorococcus sp. AG-363-P15	GCA_003278175.1	unassigned *Prochlorococcus* clade
Prochlorococcus sp. AG-363-P01	GCA_003278225.1	unassigned *Prochlorococcus* clade
Prochlorococcus sp. AG-409-I11	GCA_003215725.1	unassigned *Prochlorococcus* clade
Prochlorococcus marinus str. MIT 9211	GCA_000018585.1	*Riococcus*, LL II-III
Prochlorococcus sp. AG-402-G22	GCA_003217035.1	*Riococcus*, LL II-III
Prochlorococcus sp. MIT 0601	GCA_000760175.1	*Riococcus*, LL II-III
Prochlorococcus sp. AG-363-M21	GCA_003278305.1	*Riococcus*, LL II-III
Prochlorococcus sp. AG-363-N16	GCA_003280135.1	*Riococcus*, LL II-III
Prochlorococcus sp. AG-432-D11	GCA_003212885.1	*Prochlorococcus sensu stricto*, LL II-III
Prochlorococcus sp. AG-409-L21	GCA_003215475.1	*Prochlorococcus sensu stricto*, LL II-III
Prochlorococcus sp. AG-409-K04	GCA_003215555.1	*Prochlorococcus sensu stricto*, LL II-III
Prochlorococcus sp. AG-436-C13	GCA_003212695.1	*Prochlorococcus sensu stricto*, LL II-III
Prochlorococcus sp. AG-363-C02	GCA_003278345.1	*Prochlorococcus sensu stricto*, LL II-III
Prochlorococcus sp. AG-436-K22	GCA_003212555.1	*Prochlorococcus sensu stricto*, LL II-III
Prochlorococcus sp. AG-412-P08	GCA_003214695.1	*Prochlorococcus sensu stricto*, LL II-III
Prochlorococcus sp. AG-402-G10	GCA_003279445.1	*Prochlorococcus sensu stricto*, LL II-III
Prochlorococcus sp. AG-409-F19	GCA_003215795.1	*Prochlorococcus sensu stricto*, LL II-III

Prochlorococcus sp. AG-363-B11	GCA_003278425.1	*Prochlorococcus sensu stricto*, LL II-III
Prochlorococcus sp. AG-436-M02	GCA_003212515.1	*Prochlorococcus sensu stricto*, LL II-III
Prochlorococcus sp. MIT 0603	GCA_000760215.1	*Prochlorococcus sensu stricto*, LL II-III
Prochlorococcus marinus subsp. *marinus* str. CCMP1375	GCA_000007925.1	*Prochlorococcus sensu stricto*, LL II-III
Prochlorococcus sp. AG-409-D09	GCA_003215935.1	*Prolificoccus*, LL I
Prochlorococcus sp. AG-409-P19	GCA_003215315.1	*Prolificoccus*, LL I
Prochlorococcus sp. AG-315-I14	GCA_003283665.1	*Prolificoccus*, LL I
Prochlorococcus sp. AG-315-C08	GCA_003284185.1	*Prolificoccus*, LL I
Prochlorococcus sp. AG-315-D17	GCA_003283685.1	*Prolificoccus*, LL I
Prochlorococcus sp. AG-315-B03	GCA_003283745.1	*Prolificoccus*, LL I
Prochlorococcus sp. AG-402-P16	GCA_003216295.1	*Prolificoccus*, LL I
Prochlorococcus marinus XMU1408	GCA_003208055.1	*Prolificoccus*, LL I
Prochlorococcus sp. AG-402-K14	GCA_003216815.1	*Prolificoccus*, LL I
Prochlorococcus sp. AG-436-J02	GCA_003212625.1	*Prolificoccus*, LL I
Prochlorococcus sp. AG-363-B05	GCA_003278445.1	*Prolificoccus*, LL I
Prochlorococcus sp. AG-402-L09	GCA_003216715.1	*Prolificoccus*, LL I
Prochlorococcus sp. AG-402-A21	GCA_003279655.1	*Prolificoccus*, LL I
Prochlorococcus sp. AG-409-J03	GCA_003215695.1	*Prolificoccus*, LL I
Prochlorococcus sp. AG-311-J23	GCA_003284375.1	*Prolificoccus*, LL I
Prochlorococcus sp. AG-311-D23	GCA_003284445.1	*Prolificoccus*, LL I
Prochlorococcus sp. MIT 0801	GCA_000757865.1	*Prolificoccus*, LL I
Prochlorococcus sp. AG-424-E20	GCA_003213355.1	*Prolificoccus*, LL I

Prochlorococcus sp. AG-402-B05	GCA_003279635.1	*Prolificoccus*, LL I
Prochlorococcus marinus str. NATL2A	GCA_000012465.1	*Prolificoccus*, LL I
Prochlorococcus sp. AG-436-C14	GCA_003209755.1	*Prolificoccus*, LL I
Prochlorococcus sp. AG-402-K04	GCA_003216895.1	*Prolificoccus*, LL I
Prochlorococcus sp. AG-409-D14	GCA_003215895.1	*Prolificoccus*, LL I
Prochlorococcus sp. AG-409-A10	GCA_003216175.1	*Prolificoccus* s, LL I
Prochlorococcus sp. AG-402-I20	GCA_003216975.1	*Prolificoccus*, LL I
Prochlorococcus sp. AG-402-M18	GCA_003216595.1	*Prolificoccus*, LL I
Prochlorococcus sp. AG-402-I21	GCA_003216955.1	*Prolificoccus*, LL I
Prochlorococcus sp. AG-402-A08	GCA_003279585.1	*Prolificoccus*, LL I
Prochlorococcus sp. AG-363-A03	GCA_003280445.1	*Prolificoccus*, LL I
Prochlorococcus sp. AG-402-O21	GCA_003216375.1	*Prolificoccus*, LL I
Prochlorococcus sp. AG-402-C09	GCA_003279565.1	*Prolificoccus*, LL I
Prochlorococcus sp. AG-402-G19	GCA_003279455.1	*Prolificoccus*, LL I
Prochlorococcus sp. AG-363-G03	GCA_003280265.1	*Prolificoccus*, LL I
Prochlorococcus sp. AG-402-G06	GCA_003277985.1	*Prolificoccus*, LL I
Prochlorococcus sp. AG-402-B19	GCA_003279615.1	*Prolificoccus*, LL I
Prochlorococcus sp. AG-402-M23	GCA_003216575.1	*Prolificoccus*, LL I
Prochlorococcus sp. AG-402-P18	GCA_003216275.1	*Prolificoccus*, LL I
Prochlorococcus sp. AG-402-N21	GCA_003216435.1	unassigned *Prochlorococcus* clade
Prochlorococcus sp. AG-418-C09	GCA_003214355.1	*Eurycolium*, HL III-IV
Prochlorococcus marinus bv. HNLC1	GCA_000218705.1	*Eurycolium*, HL III-IV
Prochlorococcus marinus bv. HNLC2	GCA_000218745.1	*Eurycolium*, HL III-IV

Prochlorococcus sp. W8	GCA_000291825.1	*Eurycolium*, HL III-IV
Prochlorococcus sp. AG-402-L18	GCA_003216725.1	*Eurycolium*, unassigned *Prochlorococcus* clade
Prochlorococcus sp. AG-449-P16	GCA_003212175.1	*Eurycolium*, unassigned *Prochlorococcus* clade
Prochlorococcus sp. AG-436-O11	GCA_003212535.1	*Eurycolium*, HL VI
Prochlorococcus sp. AG-436-C05	GCA_003209795.1	*Eurycolium*, HL VI
Prochlorococcus sp. AG-363-L17	GCA_003280165.1	*Eurycolium*, HL VI
Prochlorococcus sp. AG-402-K10	GCA_003216835.1	*Eurycolium*, HL VI
Prochlorococcus sp. AG-409-A22	GCA_003216135.1	*Eurycolium*, HL VI
Prochlorococcus sp. AG-363-P06	GCA_003280025.1	*Eurycolium*, HL VI
Prochlorococcus sp. AG-418-K17	GCA_003214055.1	*Eurycolium*, HL II
Prochlorococcus sp. AG-388-A01	GCA_003279945.1	*Eurycolium*, HL II
Prochlorococcus sp. AG-347-J20	GCA_003281485.1	*Eurycolium*, HL II
Prochlorococcus sp. scB241_528O2	GCA_000634115.1	*Eurycolium*, HL II
Prochlorococcus marinus str. MIT 9107	GCA_000759855.1	*Eurycolium*, HL II
Prochlorococcus sp. AG-436-A04	GCA_003212755.1	*Eurycolium*, HL II
Prochlorococcus marinus str. MIT 9302	GCA_000759975.1	*Eurycolium*, HL II
Prochlorococcus sp. AG-418-F16	GCA_003214235.1	*Eurycolium*, HL II
Prochlorococcus sp. AG-412-C21	GCA_003214975.1	*Eurycolium*, HL II
Prochlorococcus sp. AG-424-M03	GCA_003213275.1	*Eurycolium*, HL II
Prochlorococcus marinus SCGC AAA795-J16	GCA_001180305.1	*Eurycolium*, HL II
Prochlorococcus sp. AG-347-N23	GCA_003281125.1	*Eurycolium*, HL II

Prochlorococcus marinus str. MIT 9312	GCA_000012645.1	*Eurycolium*, HL II
Prochlorococcus sp. AG-436-E22	GCA_003212615.1	*Eurycolium*, HL II
Prochlorococcus sp. AG-418-I20	GCA_003214175.1	*Eurycolium*, HL II
Prochlorococcus sp. AG-402-K21	GCA_003216775.1	*Eurycolium*, HL II
Prochlorococcus sp. AG-418-G23	GCA_003214195.1	*Eurycolium*, HL II
Prochlorococcus sp. AG-347-K18	GCA_003281365.1	*Eurycolium*, HL II
Prochlorococcus sp. AG-402-M15	GCA_003216615.1	*Eurycolium*, HL II
Prochlorococcus marinus str. GP2	GCA_000759885.1	*Eurycolium*, HL II
Prochlorococcus sp. AG-418-F08	GCA_003214295.1	*Eurycolium*, HL II
Prochlorococcus sp. AG-388-F11	GCA_003279845.1	*Eurycolium*, HL II
Prochlorococcus sp. AG-355-M18	GCA_003280725.1	*Eurycolium*, HL II
Prochlorococcus sp. AG-402-O16	GCA_003216415.1	*Eurycolium*, HL II
Prochlorococcus sp. AG-459-E08	GCA_003209425.1	*Eurycolium*, HL II
Prochlorococcus sp. AG-347-M15	GCA_003281185.1	*Eurycolium*, HL II
Prochlorococcus sp. AG-355-P07	GCA_003280575.1	*Eurycolium*, HL II
Prochlorococcus sp. AG-347-I15	GCA_003281585.1	*Eurycolium*, HL II
Prochlorococcus sp. AG-402-K22	GCA_003216755.1	*Eurycolium*, HL II
Prochlorococcus sp. scB245a_520K10	GCA_000634515.1	*Eurycolium*, HL II
Prochlorococcus sp. AG-402-C22	GCA_003279535.1	*Eurycolium*, HL II
Prochlorococcus marinus str. MIT 9201	GCA_000759955.1	*Eurycolium*, HL II
Prochlorococcus sp. AG-449-J16	GCA_003209565.1	*Eurycolium*, HL II
Prochlorococcus sp. AG-347-K15	GCA_003281415.1	*Eurycolium*, HL II

Prochlorococcus sp. HOT208_60m_813G15	GCA_002026165.1	*Eurycolium*, HL II
Prochlorococcus sp. AG-424-P23	GCA_003210135.1	*Eurycolium*, HL II
Prochlorococcus sp. AG-347-E03	GCA_003279115.1	*Eurycolium*, HL II
Prochlorococcus marinus SCGC AAA795-I06	GCA_001180265.1	*Eurycolium*, HL II
Prochlorococcus marinus str. MIT 9401	GCA_000760095.1	*Eurycolium*, HL II
Prochlorococcus sp. HOT208_60m_813O14	GCA_002025945.1	*Eurycolium*, HL II
Prochlorococcus sp. AG-449-G23	GCA_003209585.1	*Eurycolium*, HL II
Prochlorococcus sp. AG-402-N08	GCA_003216535.1	*Eurycolium*, HL II
Prochlorococcus sp. AG-418-D13	GCA_003214315.1	*Eurycolium*, HL II
Prochlorococcus sp. AG-402-L23	GCA_003216635.1	*Eurycolium*, HL II
Prochlorococcus sp. AG-412-J13	GCA_003214815.1	*Eurycolium*, HL II
Prochlorococcus sp. AG-402-N17	GCA_003216455.1	*Eurycolium*, HL II
Prochlorococcus sp. AG-418-O03	GCA_003213855.1	*Eurycolium*, HL II
Prochlorococcus sp. AG-459-O09	GCA_003211955.1	*Eurycolium*, HL II
Prochlorococcus sp. AG-402-I23	GCA_003216915.1	*Eurycolium*, HL II
Prochlorococcus sp. AG-355-J04	GCA_003280885.1	*Eurycolium*, HL II
Prochlorococcus sp. AG-347-K22	GCA_003281285.1	*Eurycolium*, HL II
Prochlorococcus sp. AG-347-E23	GCA_003281685.1	*Eurycolium*, HL II
Prochlorococcus sp. AG-347-L02	GCA_003281255.1	*Eurycolium*, HL II
Prochlorococcus sp. AG-459-D04	GCA_003212035.1	*Eurycolium*, HL II
Prochlorococcus sp. AG-418-I21	GCA_003214115.1	*Eurycolium*, HL II

Prochlorococcus sp. AG-347-M18	GCA_003281165.1	*Eurycolium*, HL II
Prochlorococcus sp. AG-347-I21	GCA_003279055.1	*Eurycolium*, HL II
Prochlorococcus sp. AG-347-I04	GCA_003281625.1	*Eurycolium*, HL II
Prochlorococcus sp. AG-347-L19	GCA_003278895.1	*Eurycolium*, HL II
Prochlorococcus marinus str. MIT 9215	GCA_000018065.1	*Eurycolium*, HL II
Prochlorococcus sp. AG-418-G18	GCA_003214215.1	*Eurycolium*, HL II
Prochlorococcus sp. AG-424-A03	GCA_003213375.1	*Eurycolium*, HL II
Prochlorococcus sp. MIT 0604	GCA_000757845.1	*Eurycolium*, HL II
Prochlorococcus sp. scB243_495D8	GCA_000634195.1	*Eurycolium*, HL II
Prochlorococcus sp. AG-402-N23	GCA_003216355.1	*Eurycolium*, HL II
Prochlorococcus sp. AG-402-K16	GCA_003216795.1	*Eurycolium*, HL II
Prochlorococcus sp. AG-355-P16	GCA_003278525.1	*Eurycolium*, HL II
Prochlorococcus sp. AG-347-G20	GCA_003281645.1	*Eurycolium*, HL II
Prochlorococcus marinus SCGC AAA795-I15	GCA_001180285.1	*Eurycolium*, HL II
Prochlorococcus sp. AG-355-J23	GCA_003278705.1	*Eurycolium*, HL II
Prochlorococcus sp. AG-347-K10	GCA_003278955.1	*Eurycolium*, HL II
Prochlorococcus sp. AG-449-O05	GCA_003212195.1	*Eurycolium*, HL II
Prochlorococcus sp. AG-355-I20	GCA_003280895.1	*Eurycolium*, HL II
Prochlorococcus sp. AG-347-L17	GCA_003278925.1	*Eurycolium*, HL II
Prochlorococcus sp. AG-455-E15	GCA_003212115.1	*Eurycolium*, HL II
Prochlorococcus sp. AG-459-A02	GCA_003212055.1	*Eurycolium*, HL II
Prochlorococcus sp. AG-442-B03	GCA_003212395.1	*Eurycolium*, HL II

Prochlorococcus sp. scB243_496M6	GCA_000635535.1	*Eurycolium*, HL II
Prochlorococcus sp. AG-436-D21	GCA_003212655.1	*Eurycolium*, HL II
Prochlorococcus sp. AG-355-B23	GCA_003280935.1	*Eurycolium*, HL II
Prochlorococcus marinus SCGC AAA795-M23	GCA_001180325.1	*Eurycolium*, HL II
Prochlorococcus sp. AG-347-K20	GCA_003281315.1	*Eurycolium*, HL II
Prochlorococcus sp. AG-670-M15	GCA_003211415.1	*Eurycolium*, HL II
Prochlorococcus sp. AG-347-L21	GCA_003278875.1	*Eurycolium*, HL II
Prochlorococcus sp. AG-347-B23	GCA_003281715.1	*Eurycolium*, HL II
Prochlorococcus sp. AG-347-L20	GCA_003281225.1	*Eurycolium*, HL II
Prochlorococcus sp. AG-442-N07	GCA_003209675.1	*Eurycolium*, HL II
Prochlorococcus sp. HOT212_60m_826P21	GCA_002026065.1	*Eurycolium*, HL II
Prochlorococcus sp. HOT208_60m_808G21	GCA_002026015.1	*Eurycolium*, HL II
Prochlorococcus sp. HOT212_60m_824C06	GCA_002026105.1	*Eurycolium*, HL II
Prochlorococcus sp. AG-355-O17	GCA_003280655.1	*Eurycolium*, HL II
Prochlorococcus sp. AG-355-N16	GCA_003278545.1	*Eurycolium*, HL II
Prochlorococcus marinus str. SB	GCA_000760115.1	*Eurycolium*, HL II
Prochlorococcus sp. AG-355-K03	GCA_003278685.1	*Eurycolium*, HL II
Prochlorococcus sp. RS04	GCA_001989455.1	*Eurycolium*, HL II
Prochlorococcus sp. HOT208_60m_813I02	GCA_002025865.1	*Eurycolium*, HL II
Prochlorococcus sp. AG-347-M23	GCA_003278855.1	*Eurycolium*, HL II

Prochlorococcus sp. AG-355-A09	GCA_003280995.1	*Eurycolium*, HL II
Prochlorococcus sp. AG-347-J06	GCA_003281525.1	*Eurycolium*, HL II
Prochlorococcus sp. AG-418-J17	GCA_003214085.1	*Eurycolium*, HL II
Prochlorococcus sp. HOT208_60m_813L03	GCA_002026145.1	*Eurycolium*, HL II
Prochlorococcus sp. scB243_495K23	GCA_000635495.1	*Eurycolium*, HL II
Prochlorococcus sp. AG-355-G23	GCA_003278765.1	*Eurycolium*, HL II
Prochlorococcus sp. scB243_498I20	GCA_000635015.1	*Eurycolium*, HL II
Prochlorococcus sp. AG-347-J21	GCA_003281455.1	*Eurycolium*, HL II
Prochlorococcus sp. AG-347-O22	GCA_003278845.1	*Eurycolium*, HL II
Prochlorococcus sp. AG-424-P16	GCA_003213255.1	*Eurycolium*, HL II
Prochlorococcus sp. HOT208_60m_808M21	GCA_002026005.1	*Eurycolium*, HL II
Prochlorococcus sp. scB245a_519A13	GCA_000634415.1	*Eurycolium*, HL II
Prochlorococcus sp. AG-355-J09	GCA_003278735.1	*Eurycolium*, HL II
Prochlorococcus sp. AG-355-N18	GCA_003278535.1	*Eurycolium*, HL II
Prochlorococcus sp. AG-402-A04	GCA_003278045.1	*Eurycolium*, HL II
Prochlorococcus sp. AG-347-K17	GCA_003281385.1	*Eurycolium*, HL II
Prochlorococcus marinus SCGC AAA795-F05	GCA_001180245.1	*Eurycolium*, HL II
Prochlorococcus sp. HOT208_60m_813B04	GCA_002025975.1	*Eurycolium*, HL II
Prochlorococcus sp. AG-347-J14	GCA_003281505.1	*Eurycolium*, HL II
Prochlorococcus sp. AG-459-N19	GCA_003209375.1	*Eurycolium*, HL II
Prochlorococcus sp. AG-355-A18	GCA_003280985.1	*Eurycolium*, HL II

Prochlorococcus sp. scB241_528N17	GCA_00063473 5.1	*Eurycolium*, HL II
Prochlorococcus marinus str. MIT 9314	GCA_00076003 5.1	*Eurycolium*, HL II
Prochlorococcus sp. scB243_495L20	GCA_00063421 5.1	*Eurycolium*, HL II
Prochlorococcus sp. AG-459-J14	GCA_00320941 5.1	*Eurycolium*, HL II
Prochlorococcus sp. AG-459-B06	GCA_00320947 5.1	*Eurycolium*, HL II
Prochlorococcus sp. AG-347-K16	GCA_00328140 5.1	*Eurycolium*, HL II
Prochlorococcus sp. AG-347-J19	GCA_00327901 5.1	*Eurycolium*, HL II
Prochlorococcus sp. AG-347-G22	GCA_00328163 5.1	*Eurycolium*, HL II
Prochlorococcus sp. AG-355-I04	GCA_00328091 5.1	*Eurycolium*, HL II
Prochlorococcus sp. AG-355-P18	GCA_00328051 5.1	*Eurycolium*, HL II
Prochlorococcus sp. AG-424-E18	GCA_00321015 5.1	*Eurycolium*, HL II
Prochlorococcus sp. AG-347-K19	GCA_00328133 5.1	*Eurycolium*, HL II
Prochlorococcus sp. AG-424-P18	GCA_00321323 5.1	*Eurycolium*, HL II
Prochlorococcus marinus str. AS9601	GCA_00001564 5.1	*Eurycolium*, HL II
Prochlorococcus sp. AG-347-I06	GCA_00328160 5.1	*Eurycolium*, HL II
Prochlorococcus sp. AG-347-I19	GCA_00327910 5.1	*Eurycolium*, HL II
Prochlorococcus marinus str. MIT 9301	GCA_00001596 5.1	*Eurycolium*, HL II
Prochlorococcus sp. AG-459-M13	GCA_00321200 5.1	*Eurycolium*, HL I
Prochlorococcus sp. AG-347-J22	GCA_00327900 5.1	*Eurycolium*, HL I
Prochlorococcus sp. AG-442-M23	GCA_00320965 5.1	*Eurycolium*, HL I
Prochlorococcus sp. AG-442-N17	GCA_00321237 5.1	*Eurycolium*, HL I
Prochlorococcus sp. AG-418-M08	GCA_00321395 5.1	*Eurycolium*, HL I

Prochlorococcus sp. AG-679-P15	GCA_003210695.1	*Eurycolium*, HL I
Prochlorococcus sp. AG-388-D03	GCA_003279875.1	*Eurycolium*, HL I
Prochlorococcus marinus str. MIT 9515	GCA_000015665.1	*Eurycolium*, HL I
Prochlorococcus sp. AG-673-M19	GCA_003211115.1	*Eurycolium*, HL I
Prochlorococcus sp. AG-402-N10	GCA_003216465.1	*Eurycolium*, HL I
OCS116 cluster bacterium AG-430-B22	GCA_003209955.1	*Eurycolium*, HL I
Prochlorococcus sp. AG-676-M04	GCA_003210895.1	*Eurycolium*, HL I
Prochlorococcus sp. AG-321-E21	GCA_003283415.1	*Eurycolium*, HL I
Prochlorococcus sp. AG-670-O11	GCA_003211335.1	*Eurycolium*, HL I
Prochlorococcus sp. AG-670-N10	GCA_003209065.1	*Eurycolium*, HL I
Prochlorococcus sp. AG-676-P15-1	GCA_003210825.1	*Eurycolium*, HL I
Prochlorococcus sp. AG-670-O17	GCA_003211315.1	*Eurycolium*, HL I
Prochlorococcus sp. AG-679-M10	GCA_003210655.1	*Eurycolium*, HL I
Prochlorococcus marinus subsp. pastoris str. CCMP1986	GCA_000011465.1	*Eurycolium*, HL I
Prochlorococcus sp. AG-335-I21	GCA_003282225.1	*Eurycolium*, HL I
Prochlorococcus sp. AG-335-A05	GCA_003282425.1	*Eurycolium*, HL I
Prochlorococcus sp. AG-686-J21	GCA_003210285.1	*Eurycolium*, HL I
Prochlorococcus sp. AG-686-P16	GCA_003208695.1	*Eurycolium*, HL I
Prochlorococcus sp. AG-686-M10	GCA_003210275.1	*Eurycolium*, HL I
Prochlorococcus sp. AG-686-O05	GCA_003210235.1	*Eurycolium*, HL I
Prochlorococcus sp. AG-679-K21	GCA_003208885.1	*Eurycolium*, HL I

Prochlorococcus sp. AG-673-L20	GCA_003211135.1	*Eurycolium*, HL I
Prochlorococcus sp. AG-676-L21	GCA_003210915.1	*Eurycolium*, HL I
Prochlorococcus sp. AG-469-F22	GCA_003211655.1	*Eurycolium*, HL I
Prochlorococcus sp. AG-388-E21	GCA_003279855.1	*Eurycolium*, HL I
Prochlorococcus sp. AG-388-A04	GCA_003279935.1	*Eurycolium*, HL I
